# Receiver Operator Characteristic (ROC) Analysis of Lipids, Proteins, DNA Oxidative Damage, and Antioxidant Defense in Plasma and Erythrocytes of Young Reproductive-Age Men with Early Stages of Type 1 Diabetes Mellitus (T1DM) Nephropathy in the Irkutsk Region, Russia

**DOI:** 10.3390/metabo12121282

**Published:** 2022-12-16

**Authors:** Marina Darenskaya, Elena Chugunova, Sergey Kolesnikov, Natalya Semenova, Isay Michalevich, Olga Nikitina, Anastasya Lesnaya, Lyubov Kolesnikova

**Affiliations:** Department of Personalized and Preventive Medicine, Scientific Centre for Family Health and Human Reproduction Problems, 664003 Irkutsk, Russia

**Keywords:** type 1 diabetes mellitus, nephropathy, oxidative stress, oxidative damage, antioxidants

## Abstract

Oxidative stress plays a leading role in the pathogenesis of diabetic nephropathy. However, many aspects of oxidative stress reactions in the initial stages of this disease are not fully understood. The men cohort is of particular interest because of the severe effects of diabetes on their urogenital system. The aim of this study is to assess the intensity of lipids, proteins, DNA oxidative damage, blood antioxidant defense enzymatic, and activity of non-enzymatic components in men with type 1 diabetes mellitus (T1DM) in the early stages of diabetic nephropathy using receiver operator characteristic (ROC) analysis. This study included eighty-nine reproductive-age men in the initial stages of diabetic nephropathy (DN) and thirty-nine age- and sex-matched individuals not suffering from glycemic disorders. The DN patients were divided into two subgroups: stage 1 patients (urinary albumin < 30 mg/day and albumin/creatinine ratio < 3 mg/mmol (*n* = 45)) and stage 2 patients (urinary albumin 30–300 mg/day and albumin/creatinine ratio 3–30 mg/mmol (*n* = 44)). Levels of oxidative damage products (conjugated dienes (CDs), thiobarbituric acid reactants (TBARs), methylglyoxal (MGO), and 8-hydroxy-2’-deoxyguanosine (8-OHdG)) and antioxidants (glutathione peroxidase (GPx), glutathione S-transferases π (GSTp), glutathione reductase (GR), copper and zinc-containing superoxide dismutase 1 (SOD-1), total antioxidant status (TAS), α-tocopherol, retinol, reduced glutathione (GSH), and oxidative glutathione (GSSG)) were estimated in plasma and erythrocytes. Oxidative damage to cellular structures (higher values of median CDs (1.68 µmol/L; *p* = 0.003), MGO (3.38 mg/L; *p* < 0.001) in the stage 1 group and CDs (2.28 µmol/L; *p* < 0.0001), MGO (3.52 mg/L; *p* < 0.001), 8-OHdG (19.44 ng/mL; *p* = 0.010) in the stage 2 group) and changes in the antioxidant defense system (lower values of TAS (1.14 units; *p* = 0.011), α-tocopherol (12.17 µmol/L; *p* = 0.009), GPx (1099 units; *p* = 0.0003) and elevated levels of retinol (1.35 µmol/L; *p* < 0.001) in the group with stage 1; lower values of α-tocopherol (12.65 µmol/L; *p* = 0.033), GPx (1029.7 units; *p* = 0.0001) and increased levels of GR (292.75 units; *p* < 0.001), GSH (2.54 mmol/L; *p* = 0.010), GSSG (2.31 mmol/L; *p* < 0.0001), and retinol (0.81 µmol/L; *p* = 0.005) in the stage 2 group) were identified. The ROC analysis established that the following indicators have the highest diagnostic significance for stage 1 diabetic nephropathy: CDs (AUC 0.755; *p* < 0.0001), TBARs (AUC 0.748; *p* = 0.0001), MGO (AUC 0.720; *p* = 0.0033), retinol (AUC 0.932; *p* < 0.0001), GPx (AUC 0.741; *p* = 0.0004), α-tocopherol (AUC 0.683; *p* = 0.0071), and TAS (AUC 0.686; *p* = 0.0052) and the following for stage 2 diabetic nephropathy: CDs (AUC 0.714; *p* = 0.001), TBARs (AUC 0.708; *p* = 0.001), 8-OHdG (AUC 0.658; *p* = 0.0232), GSSG (AUC 0.714; *p* = 0.001), and GSH (AUC 0.667; *p* = 0.0108). We conclude that changes in indicators of damage to lipids, proteins, DNA, and the insufficiency of antioxidant defense factors already manifest in the first stage of diabetic nephropathy in men with T1DM. The ROC established which parameters have the greatest diagnostic significance for stages 1 and 2 of diabetic nephropathy, which may be utilized as additional criteria for defining men with T1DM as being in the risk group for the development of initial manifestations of the disease and thus allow for substantiating appropriate approaches to optimize preventive measures.

## 1. Introduction

Diabetes mellitus (DM) is a serious medical, social, and economic problem of modern healthcare. The International Diabetes Association characterizes DM as a global pandemic since 537 million adults on the planet suffer from DM [1]. The Russian Federation (RF) is considered one of the leading countries in terms of DM prevalence [2]. The number of patients with T1DM in the RF exceeds 250,000 [3]. The most hazardous manifestations of DM are chronic vascular complications, which are the causes of the patients’ early disability and mortality [4].

Diabetic nephropathy (DN) and the resulting chronic renal failure (CRF) are the leading causes of death in patients with T1DM worldwide [5]. DN is a specific kidney lesion in DM occurring in 20.1% of patients with T1DM [2,5]. DN is characterized by various structural and functional changes in the renal structures that underlie the progressive decline in renal function up to the terminal renal failure [6,7].

DN is diagnosed by determining the level of albuminuria and the albumin–creatinine ratio [5,8]. However, it has been shown that changes in kidney tissues in patients with DM already occur in conditions of normal albumin excretion with urine, and the detection of albuminuria indicates the presence of sclerosis in 20–25% of nephrons [9,10]. By the time persistent albuminuria (proteinuria) occurs, 50–70% of the renal tissue is already sclerosed [11]. In this regard, early detection of potentially reversible damage in the kidneys is critical. As a result, the elucidation of the mechanisms that contribute to these changes in the preclinical stages of DN, and their corresponding correction, remain topical issues that deserve the close attention of specialists [12,13,14].

It is also unambiguously recognized that mitochondrial dysfunction contributes to the development and progression of DN [15]. Mitochondria are known to be the main suppliers of reactive oxygen species (ROS), the increased accumulation of which leads to the development of oxidative stress (OS) [16]. OS is defined as an imbalance between ROS production and the efficiency of enzymatic links of antioxidant defense [17,18]. Intense OS causes damage to cellular components and may be characterized by an increase in oxidatively modified proteins, lipids, and nucleic acids [19,20].

The interaction of ROS with cellular components—lipids, proteins, and DNA—ultimately results in modification of their structure, and these modifications can persist in DM patients for a prolonged period, even after achieving normoglycemia [21,22,23,24,25]. This phenomenon underlies the so-called “metabolic memory” mechanism that is based upon the products of oxidative damage [22,26]. The latter accumulate and persist in the vessels for extended periods, which, together with additional pathogenetic mechanisms of kidney damage, leads to the serious dysregulation of vital processes [4,22].

In spite of research efforts, the role of OS in the progression of DN remains unclear. According to Casanova et al. 2021 [27], future studies should be optimized by comparing data on renal function that include the early stages of the disease and OS. It is now known that the nature of metabolic responses in T1DM depends on many factors, including the age and sex of patients [26,28]. The development of T1DM in young reproductive-age persons, particularly in men, increases the importance of preventing and treating its associated complications due to the high risk of reproductive disorders [29,30]. In addition, in a study by Perkins, high HbA1c levels and men were found to be the most significant factors independently associated with the progression of DN [31]. There is still insufficient knowledge about the activity of OS reactions in men with T1DM depending on the level of albuminuria. Therefore, there is particular interest in this cohort of patients.

Based on the above background, the aim of this study was to assess the intensity of lipids, proteins, DNA oxidative damage, blood antioxidant defense enzymatic, and the activity of non-enzymatic components in men with T1DM in the early stages of DN using ROC analysis.

## 2. Materials and Methods

An informed consent form was obtained from all participants in the study in accordance with the World Medical Association Declaration of Helsinki (1964, 2013 ed.). No additional interventions were provided. The personal data of patients were not disclosed during the study. The research was approved by the Biomedical Ethics Committee at the Scientific Centre for Family Health and Human Reproduction Problems, and Irkutsk Regional Clinical Hospital (IRCH), Russia (permission number 8.2, dated 2 November 2018).

### 2.1. Patients

Eighty-nine reproductive-age men (average age 31.73 ± 2.59 years) with T1DM and a poor glycemic profile (main group) were observed from November 2018 to December 2021 at the Endocrinology Department of the IRCH, Russia. Inclusion criteria: In the main group, men of age 18–40 years old, place of residence in the Irkutsk region, informed consent to participation in the study, diagnosis of T1DM, glomerular filtration rate (GFR) > 60 mL/min/1.73 m^2^. Exclusion criteria: type 2 of DM (T2DM), macrovascular complications, proteinuria or renal failure, other endocrine diseases, concomitant somatic pathology. Based on the classification [32], the main group was divided into two subgroups: stage 1 patients (urinary albumin < 30 mg/day and ratio of albumin/creatinine < 3 mg/mmol) (n = 45 patients) (Group 1) and stage 2 patients (urinary albumin—30–300 mg/day and ratio of albumin/creatinine—3–30 mg/mmol) (n = 44 patients) (Group 2). Material from all patients was collected prior to the commencement of treatment. An endocrinologist (E.C.) examined both groups.

Classification of diabetes (World Health Organization (WHO, 1999), criteria for a diagnosis of DM (WHO, 1999–2018), and medical care algorithms for patients with DM were used in the study.

All patients received long-acting and ultra-short-acting human insulin taking into account individual treatment plans. Body mass index (BMI) was calculated according to the standard method (weight in kilograms divided by the square of height in meters).

Thirty-nine individuals not suffering from glycemic disorders applying for a preventive examination at the IRCH, Russia, from November 2018 to December 2021 made up the control group (average age 29.7 ± 4.6 years old). This group was matched to the clinical groups for sex and age. Inclusion criteria for the control group: men of age 18–40 years old and normal level of glucose. Exclusion criteria for the control group: hereditary predisposition to DM and acute or exacerbation of chronic diseases at the time of the examination.

### 2.2. Blood and Urinary Collection

The S-monovette dipotassium ethylene diamine tetraacetic acid (K3-EDTA) blood collection system (Sarstedt, Germany) was used for venous blood collection (in volume 10 mL). The analysis was conducted after overnight rest of subjects, on an empty stomach, between 8.00 and 9.00 a.m. Research did not involve using food recall data and food records before taking blood from respondents.

Immediately after collection, blood was centrifuged at 1500× *g* for 10 min at 40 °C to separate the plasma from the erythrocytes. Plasma was taken, and the erythrocytes were washed three times in cold saline solution (0.9% NaCl, *w*/*v*). Then, the erythrocytes were hemolyzed by adding 9 volumes of cold 50 mM phosphate buffer of pH 7.4 (*v*:*v*) [33]. Samples were kept frozen at the temperature of −80 °C until use in oxidative damage product and antioxidant system parameter evaluation.

The first morning, urine (middle portion) was collected for analysis of creatinine and albumin/creatinine ratio. Before collecting urine, a thorough toilet of the external genital organs was carried out. On the eve of the study, it is forbidden to eat vegetables and fruits that can change the color of urine, and medications are not taken. Urine collection was carried out only in a sterile container. To collect daily urine, in the morning in the interval from 6:00 to 8:00 h, the bladder was emptied. During the day, all urine was collected in a special container with a volume of at least 2 L. The last portion was collected exactly at the same time when the collection was started the day before (the start and end times of the collection were noted). At the end of daily urine collection, the sample was thoroughly mixed. The volume of daily urine (diuresis) was measured and recorded. Using a device for transferring the sample from the container, urine was collected into a vacuum tube. The tube was marked (sample number in accordance with the referral form) and transferred to the laboratory, indicating the total volume of urine excreted per day.

### 2.3. Biochemical Analysis

The performed analysis included assessment of glycosylated hemoglobin (HbA1c), average daily hyperglycemia, blood and urinary creatinine, blood and urinary albumin, total protein (TP), blood urine, albumin/creatinine ratio, GFR, total cholesterol (TC), high-density lipoprotein cholesterol (HDL), triacylglycerides (TG), oxidative damage products (CDs, TBARs, MGO, 8-OHdG) as well as TAS, antioxidant enzymes (GPx, GSTp, GR, SOD-1), and non-enzymatic antioxidants (α-tocopherol, retinol, GSH, GSSG).

### 2.4. Biochemical Parameters

HbA1c in erythrocytes was measured by high-performance liquid ion exchange chromatography (D-10 analyzer (Bio-Rad, Hercules, CA, USA). The glucose oxidase method was used for measuring capillary glucose. The average daily hyperglycemia was determined.

The serum lipid content (TG, TC, and HDL) was determined using commercial kits (BioSystems, Spain) and a biochemical analyzer (Synchron SH9 Pro, Beckman Coulter, Brea, CA, USA). The levels of LDL were calculated using the Friedewald formula [34]: LDL = TC − (HDL + VLDL)). The level of VLDL equaled TG/2.2.

Methods used for kidney damage determination in the early studies included a GFR calculation, urinary albumin and ratio of albumin/creatinine. These were determined on a biochemical analyzer (Synchron SH9 Pro, Beckman Coulter, Brea, CA, USA) using the immunoturbidimetric method. The Chronic Kidney Disease Epidemiology Collaboration (CKD-EPI) formula was used for calculating GFR (mL/min/1.73 m^2^).

### 2.5. Plasma Oxidative Damage Products

Plasma was analyzed to determine the levels of oxidative damage products (CDs, TBARs, MGO, 8-OHdG).

The CD levels were analyzed by absorbance of plasma heptane extracts at λ = 232 nm [35]. To 0.2 mL of serum was added 8 mL of a mixture of heptane–isopropanol (1:1). The tubes were shaken for 15 min on a laboratory shaker LT-2 (Czech Republic). Next, 1 mL of HCl (PH = 2.0) was added, quickly shaken and left for 10–15 min. The upper layer was selected for the study. The concentration was calculated according to the formula: $\frac{D_{232}\times V_{ex}}{V_{b}}=D_{232}\times 20,$ per 1 mL of serum, where the measured *D*_232_—value of optical density (OD), *V_ex_*—4 mL—final volume of heptane extract, *V_b_*—0.2 mL—the volume of blood serum. The CDs content was expressed in µmol/L.

Commercial kits (Agat, Moscow, Russia) detected the TBAR levels. In this method, LPO products form a colored complex with thiobarbituric acid (TBA), which can be extracted with butanol. Plasma TBAR levels were determined using TBA reaction followed by detection of the intensity of fluorescence (at λ = 515 nm (excitation) and λ = 554 nm (emission)). TBAR content is expressed in μmol/L. The CDs and TBAR measurements were carried out on the Shimadzu RF-1501 spectrofluorophotometer (Tokio, Japan).

Levels of MGO, a serum carbonyl stress indicator, were determined using the commercial Human MGO enzyme-linked immunosorbent assay (ELISA) Kit (Wayne, USA). Sample MGO concentrations were determined by comparing the OD of MGO value of the samples to those of the standard curve. MGO concentration is expressed in mg/L.

The 8-OHdG concentration was determined with an Assay Design DNA Damage ELISA kit (USA), as previously shown [36]. The kit is based on a fast and sensitive competitive enzyme immunoassay and is designed to determine 8-OHdG in urine, serum, and saliva samples. The 8-OHdG concentration is expressed in ng/mL. The enzyme immunoassays (MGO and 8-OHdG) were performed on a MultiSkan ELX808 microplate reader (Biotek, Winooski, VT, USA) at 450 nm.

This work was carried out using the equipment of the Centre of Collective Usage, Center for the Development of Progressive Personalized Health Technologies, Scientific Centre for Family Health and Human Reproduction Problems, Irkutsk.

### 2.6. Plasma and Erythrocytes Antioxidants

GPx, GSTp, GR, SOD-1, GSH, and GSSG were estimated in erythrocytes, whereas TAS, α-tocopherol, and retinol were analyzed in plasma.

The activity of GPx, GR, and SOD-1 in erythrocytes was determined using commercial RANSOD kits (Randox Laboratories Ltd., Crumlin, UK) and a microplate reader (MultiSkan ELX808, Biotek, Winooski, VT, USA) as instructed by the manufacturer. The enzyme activities were expressed in equivalent units.

GPx determining method based on GPx catalysis the oxidation of GSH by cumene hydroperoxide, and its activity corresponds to the decrease in absorbance at λ = 340 nm.

The method for determining GR activity is based on GR catalyzing the reduction of GSSG in the presence of NADPH, which is oxidized to NADP^+^, and activity is measured as the decrease in absorbance at λ = 340 nm.

The basis of the SOD-1 method involves xanthine and xanthine oxidase (XOD), which are employed to generate superoxide radicals that react with 2-(4-iodophenyl)-3-(4-nitrophenyl)-5 (phenyl) tetrazolium chloride to form a red formazan dye. GSH and GSSG levels were determined according to a fluorimetric method [37] on the Shimadzu RF-1501 spectrofluorophotometer (Tokio, Japan). The essence of the technique lies in the ability of GSH to specifically react with ortho-phthalaldehyde (OPA) at pH 8.0 to form a fluorescent product that can be activated at λ = 350 nm and has an emission peak at λ = 420 nm. The determination of GSSG was similarly carried out with OPA but in a more alkaline medium (pH 12.0) and with the addition of N-ethylmalienite to the samples to prevent the oxidation of GSH into GSSG. The conditions for recording fluorescence were identical for both. The measurements were carried out at λ = 350 nm for excitation and λ = 420 nm for emission. The GSH and GSSG concentrations are expressed in mmol/L.

The GSTp levels were detected by immunoenzymometric assay using ELISA kits (Cloud-Clone Corp., Katy, TX, USA) and a microplate reader (MultiSkan ELX808, Biotek, Winooski, VT, USA) as instructed by the manufacturer. Concentrations are expressed in ng/mL.

TAS was determined using commercial kits, RANSOD (Randox, Crumlin, UK). 2,2’-Azino-bis (3-ethylbenzothiazoline-6-sulfonic acid) or ABTS was incubated with peroxidase (metmyoglobulin) and H_2_O_2_ to produce the radical cation ABTS. The measurements were carried out on a BTS-350 spectrofluorophotometer (Barcelona, Spain) at λ = 600 nm. TAS is expressed in conventional units.

Blood plasma vitamin (α-tocopherol and retinol) concentrations were spectrophotometrically detected (described by Chernjauskene et al. [38]) on a Shimadzu RF-1501 spectrofluorophotometer (Tokio, Japan). The method for determining the concentrations of α-tocopherol and retinol involves the removal of substances that prevent the determination by saponification of samples in the presence of large amounts of ascorbic acid and the extraction of unsaponifiable lipids with hexane, followed by fluorimetric determination of the content of α-tocopherol and retinol. At the same time, α-tocopherol has intense fluorescence with a maximum of excitation at λ = 294 nm and emission at λ = 330 nm; retinol at λ = 335 nm and λ = 460 nm, respectively.

### 2.7. Statistical Analysis

The results were calculated in STATISTICA 10.0 (Stat-Soft Incorporated, Tulsa, OK, USA). The visual–graphical method and Kolmogorov–Smirnov agreement criterion (with Lilliefors and Shapiro–Wilk correction) were used to determine the normality of distribution of the quantitative parameters. The nonparametric Mann–Whitney (U-test) method was used due to abnormal distribution of the data in the study groups. Data are presented as the median (quartile 1 (Q1); quartile 3 (Q3)). The diagnostic value and optimal cut-off levels of oxidative damage products and antioxidants were determined based on the analysis of surface area under the ROC curve, also known as the area under curve (AUC).

The significance level was assumed to be *p* < 0.05.

## 3. Results

### 3.1. Clinical Findings

The clinical characteristics of all participants with T1DM are summarized in Table 1.

Patients of Groups 1 and 2 showed significant differences compared with the control group in terms of HbA1c and average daily hyperglycemia parameters (*p* < 0.05). Groups 1 and 2 did not differ (*p* > 0.05), except for average daily glycemia, which was 1.17 times higher (*p* = 0.02) in Group 2 (Table 1).

### 3.2. Biochemical Characteristics

According to the findings, Group 1 had higher TG (*p* = 0.007) and VLDL (*p* = 0.007) and lower creatinine (*p* < 0.0001) and TP (*p* = 0.001) values relative to controls (Table 2).

Group 2 also had higher TC (*p* = 0.001), TG (*p* = 0.022), VLDL (*p* = 0.022), and lower creatinine (*p* < 0.0001) values relative to controls (Table 2). Albumin levels were higher (*p* < 0.0001) in Group 2 than in Group 1 (Table 2). No statistically significant differences (*p* > 0.05) were found in the other indices in the studied groups (Table 2).

Analysis of the biochemical parameters in the groups showed that Group 1 had higher GFR values compared to the control group (*p* < 0.0001) (Table 3).

Group 2 had lower urinary creatinine values (*p* = 0.003) and higher urinary albumin levels (*p* < 0.0001) and albumin/creatinine ratios (*p* < 0.0001) compared with the control group (Table 3). Intergroup differences were characterized by higher urinary albumin (*p* < 0.0001) and albumin/creatinine ratio (*p* < 0.0001) and decreased GFR (*p* = 0.024) in the urine of Group 2 compared with Group 1 patients (Table 3).

### 3.3. Oxidative Damage Products

It was noted that the CD levels were higher in T1DM patients than the control for both Group 1 (*p* = 0.003) and Group 2 (*p* < 0.0001) (Table 4).

At the same time, the CD levels were 1.32 times higher (*p* = 0.011) in Group 2 than Group 1 (Table 4). The final TBAR products showed another trend compared with controls, with lower values in Groups 1 (*p* = 0.023) and 2 (*p* = 0.023) (Table 4). The MGO concentrations were statistically significantly higher in both Groups 1 (*p* < 0.001) and 2 (*p* < 0.001) than controls (Table 4). When comparing the values of 8-OHdG in the groups of T1DM patients, statistically higher values of this parameter were found in Group 2 compared with controls (*p* = 0.010) (Table 4). Comparison of the results between both groups also showed that values of 8-OHdG (*p* = 0.010) are elevated in Group 2 patients (Table 4).

### 3.4. Total Antioxidant Status, Enzymatic and Non-Enzymatic Antioxidants

The assessment of TAS levels showed a decrease in their values in Group 1 (*p* = 0.011) relative to the control group (Table 5).

Lower values for α-tocopherol concentration were noted in both Group 1 (*p* = 0.009) and Group 2 (*p* = 0.033) patients (Table 5). Retinol levels, however, increased in Groups 1 (*p* < 0.001) and 2 (*p* = 0.005) compared with the control group (Table 5). Regarding glutathione system parameters, statistically significant differences in GPx activity were found in the form of its reduced values (*p* = 0.0003) in Group 1 relative to the control group (Table 5), which were lower for Group 2 (*p* = 0.0001) along with elevated values of GR (*p* < 0.001), GSH (*p* = 0.010), and GSSG (*p* < 0.0001) (Table 5). Comparison between Groups 1 and 2 revealed a statistically significant difference in GSH and GSSG levels, where the values were higher in Group 2 relative to Group 1 (*p* = 0.027 and *p* = 0.003) (Table 5).

### 3.5. ROC-Analysis

In this study, we carried out ROC analysis of the discriminative abilities of OS and AOD biomarkers in the diagnosis of patients with T1DM of Groups 1 and 2. For the ROC analysis, all indicators were examined to select the most significant ones. The usefulness of redox parameters and oxidative damage products in T1DM of Group 1 in comparison with those of the control group and Group 2 patients in comparison with Group 1 are presented in Table 6.

ROC analysis shows the diagnostic significance of CDs (AUC 0.755; *p* < 0.0001), TBARs (AUC 0.748; *p* = 0.0001), MGO (AUC 0.720; *p* = 0.0033) for Group 1 compared with controls. For Group 2, the diagnostic significance was similar to Group 1 in terms of CDs (AUC 0.714; *p* = 0.001) and TBARs (AUC 0.708; *p* = 0.001) parameters (Table 6). However, in this case, the parameter 8-OHdG (AUC 0.658; *p* = 0.0232) was of greater significance (Table 6).

In the AOD system, the significance of the parameters for Group 1 was identified for retinol (AUC 0.932; *p* < 0.0001), GPx (AUC 0.741; *p* = 0.0004), α-tocopherol (AUC 0.683; *p* = 0.0071), and TAS (AUC 0.686; *p* = 0.0052) (Table 6). For Group 2, regarding the AOD system parameters, significance was noted only for GSSG (AUC 0.714; *p* = 0.001) and GSH (AUC 0.667; *p* = 0.0108) (Table 6).

**Figure 1:**
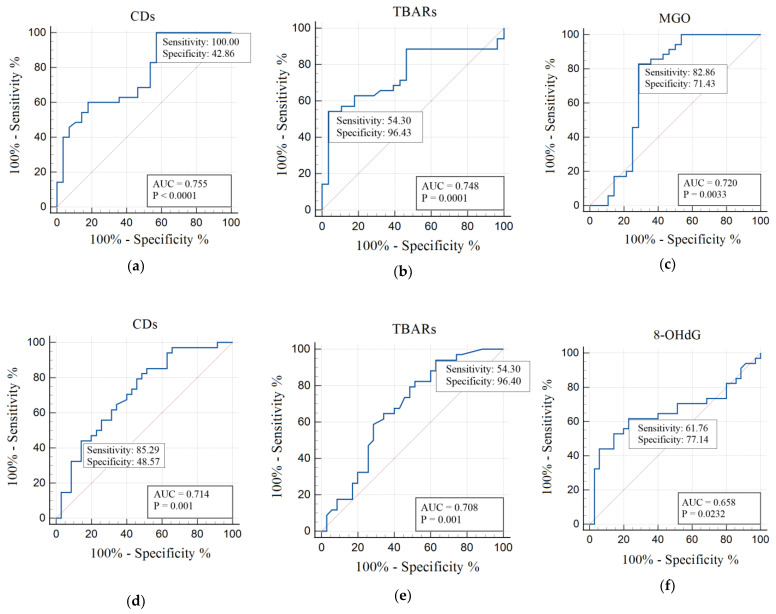
ROC analysis of oxidative damage products in Group 1 men compared with those in the control group (**a**–**c**) and in Group 2 men in comparison with Group 1 (**d**–**f**). Abbreviations: AUC, area under curve; CD, conjugated dienes; TBARs, thiobarbituric acids reactants; MGO, methylglyoxal; 8-OHdG, 8-hydroxy-2-deoxyguanosine; *p*, statistically significant differences.

**Figure 2:**
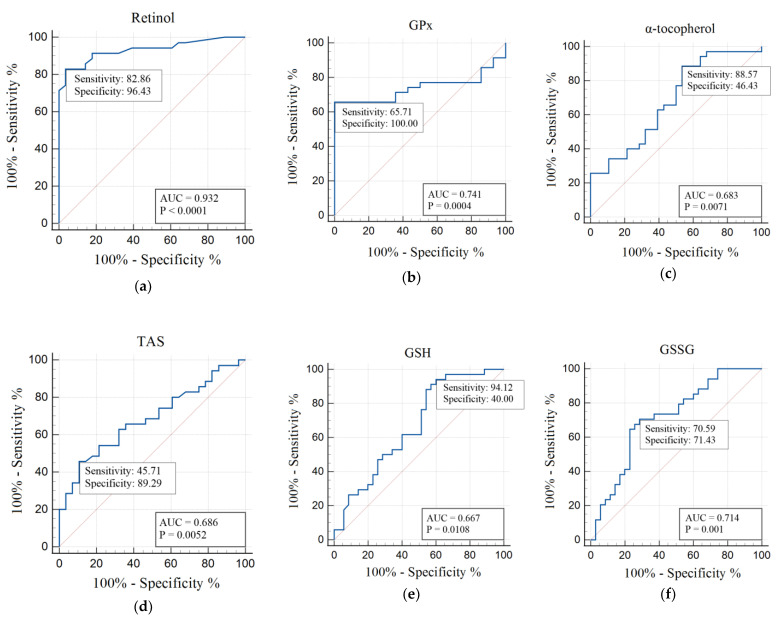
ROC analysis of total antioxidant status, enzymatic and non-enzymatic antioxidant levels in Group 1 men compared with those in the control group (**a**–**d**) and in Group 2 men in comparison with Group 1 (**e**,**f**). Abbreviations: AUC, area under curve; GPx, glutathione peroxidase; GSH, reduced glutathione; GSSG, oxidative glutathione; *p*, statistically significant differences; TAS, total antioxidant status.

Higher sensitivity values in Group 1 were noted for indicators of CDs (100%), MGO (82.86%), α-tocopherol (88.57%), retinol (82.86%) and GPx (65.71%) (Figure 1 and Figure 2).

In Group 1, higher sensitivity values were showed for GSH (94.12%), CDs (85.29%), GSSG (70.59%), and 8-OHdG (61.76%) (Figure 1 and Figure 2).

## 4. Discussion

The clinical and anamnestic examination of patients revealed that Groups 1 and 2 were comparable for a number of parameters; however, there were intergroup differences in urinary albumin levels and albumin/creatinine ratios that enable the T1DM patients to be divided into two groups. Furthermore, Group 1 patients had decreased creatinine levels and increased GFR, which reflect the tendency toward hyperfiltration in T1DM patients with normal urinary albumin excretion. These processes may be associated with nephropathy. Moreover, these results are consistent with those of Gounden et al. (2022) [39] and Soliman et al. (2022) [40], who reported impaired renal function and nephrosis together with changes in these parameters in T1DM patients. Changes in lipid metabolism parameters in patients tended to increase atherogenic cholesterol fractions in both groups, which are negative factors for the course of DM. Thus, abnormalities in quantitative lipoprotein levels were noted in T1DM patients with poor glycemic control, including increased TG and LDL levels [41,42]. Alrasheed (2022) noted that T1DM patients with albuminuria have significant abnormalities, such as elevated TG, LDL, and plasma apolipoprotein B levels as well as decreased HDL levels [43]. Al-Bayati et al. (2014) found that serum lipids are associated with the progression of nephropathy in T1DM [44]. Vergès (2020) noted that T1D patients with overt albuminuria show significant quantitative lipoprotein abnormalities [45]. Weldegiorgis et al. (2022) showed that elevated TG and reduced HDL levels are independently associated with the onset of advanced CKD [46]. In the FinnDiane study (Sigfrids, 2022), diabetic kidney disease (DKD) progression was assessed in terms of remnant cholesterol and apolipoprotein C-III (apoC-III), both key components of the triglyceride-rich lipoprotein metabolism [47]. It was reported that triglyceride-rich lipoprotein metabolism appears to be implicated in the development and progression of DKD. Li et al. (2022) revealed that the combination of TC/HDL ratio and the logarithm-transformed urinary albumin/creatinine ratio had significant predictive value for the progression of CKD [48]. Furthermore, in our study, we observed increased fasting blood glucose levels in all groups of T1DM patients compared with the control, indicating uncontrolled blood glucose levels in the patients. This factor, along with others, represent the main reasons for the damaging effect on the function of the renal apparatus [49]. Papadopoulou-Marketou (2018) reported that the development of microalbuminuria is associated with poor glycemic control, hyperlipidemia, smoking, oxidative stress, and accumulation of advanced glycation end products (AGEPS) [50]. 

In our study, we found that the values for the parameters of the primary LPO products, CDs, were higher in both clinical groups of T1DM patients. Most studies confirm that OS is a factor linking the main pathways involved in the initiation and progression of DN [20,26,28,51]. An increase in primary LPO products in the genesis of DN is evidenced by numerous studies [16]. Popykhova et al. (2021) presented data supporting the informativeness of the combined determination of primary products of OS, immunoinflammatory factors, vascular endothelial growth factor, and podocyte damage markers in the occurrence and development of DN [52]. A number of studies have indicated that a slight increase in ROS (in particular, lipid hydroperoxides (CDs)) above the physiological limit can induce significant conformational changes in lipids, proteins, carbohydrates, and nucleic acids, which leads to distorted interactions of the cellular functions in the renal structures [53,54]. The work of Ricciardi et al. (2021) showed that increased oxidative stress, inflammation, cell apoptosis, and tissue fibrosis drive the relentless, progressive loss of kidney function, affecting both the glomerular filtration barrier and the renal tubulointerstitium [55]. Our study obtained data on lower values of TBARs in the studied groups. In contrast, a number of studies have shown an increase in the values of lipid peroxidation end products in DN [20,23,56]. Pestana et al., however, found no significant differences when studying this parameter [57].

Elevated values for the carbonyl stress indicator, MGO, were observed in men with T1DM in both Groups 1 and 2. MGO is considered to be an important biomarker for complications of diabetes because of its close association with the glycation processes, β-cell dysfunction, and insulin resistance [52]. MGO covalently modifies DNA, RNA, and protein, forming advanced glycation end products (AGEPS) [23,58]. AGEPS accumulate, decompose slowly, and persist for a long time in the vascular bed, even after euglycemia is reached, which has been dubbed the “metabolic memory” mechanism [59]. Currently, a large number of studies are devoted to this phenomenon, including in the development of DN [22,26,59]. Fotheringham et al. (2022) noted that the kidney, as a major site for AGEP clearance, is particularly vulnerable to AGE-mediated damage, and increases in circulating AGEs are aligned with the risk of CKD and all-cause mortality [60]. Hirakawa (2017) noted that early progression of DN correlates with MGO-derived advanced glycation products [22]. The authors confirmed that an increase in the products in the early stages of DN affect the renin–angiotensin system and transforming growth factor-beta (TGF-β) signaling, causing chronic inflammation and glomerular and tubular hypertrophy [22]. Chernikov et al. (2017) found that increased levels of AGEP are closely related to various structural and functional changes characteristic of DN [59]. There is evidence that MGO is the most reactive among AGEPS due to its direct involvement in causing impairments in insulin secretion and function as well as in signal transduction processes [21]. Wu et al. (2021) showed that hyperglycemia-related AGEP formation plays a central role in the pathogenesis of diabetic kidney disease [61]. The authors established that the activation of AGE-mediated receptors for AGEs (RAGEs) could evoke nicotinamide adenine dinucleotide phosphate oxidase-induced reactive oxygen and nitrogen species production, subsequently giving rise to oxidative stress in DKD and the aging kidney [61,62].

Our study also reveals an increase in 8-OHdG in Group 2, in comparison with both the control and Group 1. The 8-OHdGs are modified nucleoside bases that are a product of oxidative DNA damage that are removed through the excision of oxidized guanosine from mitochondrial and nuclear DNA as part of the base excision and repair system [24]. It is now known that DNA oxidation is associated with a wide range of types of damage, including cell aging and apoptosis [54]. Sanchez et al. (2018) tested the associations between 8-OHdG concentration and urinary albumin concentration or GFR at baseline, and the risk of end-stage renal disease or all-cause mortality during 6 years of follow-up in two prospective cohorts of participants with T1DM. They established that higher plasma concentrations of 8-OHdG are independently associated with an increased risk of kidney disease in individuals with T1DM, so this marker can be used to evaluate the progression of diabetic kidney disease [24]. Our results are also consistent with Daehn (2018) [63] and Qi et al. (2017) [25], who found increased oxidative damage in the glomerular endothelial cells (GECs) of DN patients, as urinary excretion of damaged oxidized DNA (8-OHdG) was significantly increased in patients with progressing DN. Soliman et al. (2022) [40] showed increased values of this parameter in patients in the initial stages of DN, along with reduced values of telomere length. The results of studies in animals have shown that transforming growth factor-beta 1 levels, 8-hydroxy-2′-deoxyguanosine expression, and histopathological changes in renal tissue are increased in streptozotocin-induced diabetic rats [64]. Increased mitochondrial DNA lesions that were exclusively localized to GECs were reported in diabetic D2 mice after 3 weeks of diabetes, and these accumulated over time in addition to increased urine secretion of 8-oxo-deoxyguanosine [25]. Qi et al. (2017) found increased mitochondrial DNA products in the GECs to be responsible for early endothelial injuries and diabetes-induced podocyte depletion [25,27]. Casalena et al. (2020) demonstrated, in mouse models of DKD, that GECs become dysfunctional and pathological to neighboring podocytes due to increased levels of mitochondrial superoxide in GEC and increased frequencies of DNA lesions (8-oxoguanosine) [27]. Thus, increased levels of 8-OHdG in the blood of Group 2 patients may reflect the potential role of oxidative DNA damage in the development of DN.

As a rule, we noted changes in the form of higher values of primary LPO products, the carbonyl stress parameter, and occurrence of DNA damage when the AOD system was not able to neutralize the toxic effect of ROS. We observed decreased values of TAS in Group 1 as well as α-tocopherol in both clinical groups. TAS reflects the total activity of peroxidation inhibitors and includes numerous enzymatic and non-enzymatic factors as well as low molecular weight compounds [26]. A decrease in this parameter undoubtedly has negative effects on the state of the AOD system in T1DM patients. Similar changes in relation to TAS in patients in the initial stages of DN have been identified by Ramachandrayya et al. (2022) [65] and Chen et al. (2020) [66]. Tabur et al. (2015) showed that TAS is lower in microalbuminuric and normoalbuminuric groups with DM compared with the control [67]. Multiple regression analysis was used to detect the significant association between microalbuminuria progress and oxidative stress and urotensin-II levels in diabetic individuals [67].

The α-tocopherol concentration showed lower values in both Groups 1 and 2. Vitamin E (α-tocopherol) is a phenolic-type compound that has demonstrated effects from the subcellular to the organismal level [68,69]. It is known that reduced vitamin E concentration is associated with the development of diabetes complications [26] and that antioxidant supplements play a positive role in the prevention of macroalbuminuria and apparent nephropathy in the early phase of microalbuminuria [66]. It is likely that vitamin E influences renal function in different biochemical ways within the context of DN [70]. It was revealed that the effectiveness of vitamin E in various cellular targets, such as podocytes, endothelial cells, and mesangial cells [71], is correlated with an increase in the levels of pro-inflammatory mediators in blood serum. Vitamin E supplements have been shown to reduce both renal interstitial fibrosis and tubule epithelial cell apoptosis [70]. The results of a meta-analysis suggest that short-term treatment with antioxidant vitamins can benefit patients with diabetes and albuminuria in terms of kidney function and systolic pressure; yet, such a treatment has no significant effect on glucose and lipid metabolism [66]. In multivariate regression analyses, Xu et al. (2016) established that serum α-tocopherol levels are directly and strongly associated with insulin sensitivity index values [72]. Thus, the insufficiency of this antioxidant can significantly affect the course of DN.

In our study, we noted a decrease in GPx activity in Group 1. In Group 2, there was a decrease in GPx activity as well as higher values of GR and reduced and oxidized glutathione fractions. The data in the literature on the activity of antioxidant enzymes in the initial stages of nephropathy are quite contradictory [26,68,73], which may be due to a number of factors (glycemic control level, DM duration, and concomitant complications) [74]. Some researchers point out that there are no differences in GR and GPx activity in T1DM patients compared with controls [75]. However, reduced values of GPx were recorded in studies by Rajeshwari et al. [71]. Insufficient activity of this enzyme may indicate reduced mechanisms of phospholipid and fatty acid hydroperoxide utilization through glutathione oxidation [76]. The most likely explanation for the increased activity of the glutathione system (GR, GSH, GSSG) in Group 2 patients may be that these components participate in the mechanisms of MGO detoxification through the glycosylase system [21,22]. The activity of glutathione system enzymes is usually reduced in DM, and GSH works as a catalyst that binds to MGO to form hemithioacetal for reaction with glyoxylase-1 [21]. GSH is also regulator of many processes in the cell, including gene expression, DNA synthesis, proteolysis, cell proliferation and apoptosis, cytokine synthesis and immune defense, mitochondrial function regulation, and oxidative status [76]. However, we observed insufficient activity of the glutathione system components in Group 2, which is likely due to the continued increase in MGO in this case.

ROC analysis is often used to highlight the diagnostic significance of individual indicators [77]. Using ROC analysis in our study, we established that the indicators CDs, TBARs, MGO, retinol, GPx, α-tocopherol, and TAS had the highest diagnostic significance for Group 1 and CDs, TBARs, 8-OHdG, GSSG, and GSH had the highest diagnostic significance for Group 2. The AUC areas had increased values for these indicators, which was statistically significant. The AUC maximum for Group 1 had CDs, TBARs, MGO compared with controls. For Group 2, the AUC maximum had CDs, TBARs and 8-OHdG. In the AOD system, AUC maximum for Group 1 was identified for retinol, GPx, α-tocopherol, and TAS. For Group 2, AUC maximum was noted only for GSSG and GSH. Higher sensitivity values in Group 1 were noted for CDs, MGO, α-tocopherol, retinol and GPx; in Group 2 higher sensitivity values were showed for GSH, CDs, GSSG, and 8-OHdG.

This indicates that of the studied parameters, lipid peroxidation products and MGO, as well as antioxidant factors, are the most effective markers for diagnosing oxidative damage of biostructures in patients with stage 1 nephropathy compared to other indicators. For stage 2, other indicators already found to be associated, along with lipid peroxidation products, may have important prognostic value.

## 5. Conclusions

We conclude that changes in parameters of damage to lipids, proteins, DNA, and insufficiency of antioxidant defense factors already manifest in the first stage of DN in men T1DM. The ROC analysis established the parameters that have the greatest diagnostic significance for stages 1 (CDs, TBARs, MGO, retinol, GPx, α-tocopherol, and TAS) and 2 (CDs, TBARs, 8-OHdG, GSSG, and GSH) of DN, which may be used as additional criteria for including men with T1DM in the risk group for the development of initial manifestations of the disease and thus substantiating appropriate approaches to optimize preventive measures.

## Figures and Tables

**Table 1:** Clinical characteristics of the men with T1DM (Me, [Q1; Q3]).

Characteristics	Group 1	Group 2	Control Group
Age, years	27.00 [22.00; 34.00]	30.00 [27.00; 35.00]	29.50 [25.00; 34.00]
T1DM duration, years	7.00 [3.00; 10.00]	7.28 [3.00; 11.00]	
HbA1c, %	10.20 [8.70; 11.30] *	11.10 [9.30; 13.40] *	4.87 [3.80; 5.20]
BMI, kg/m^2^	21.10 [20.30; 23.70]	21.40 [19.30; 24.50]	21.50 [20.60; 22.40]
Average daily hyperglycemia, mmol/L	10.50 [9.10; 12.70] *	12.30 (10.30; 14.80] *,#	4.90 [3.90; 5.20]

Abbreviations: BMI, body mass index; HbA1c, glycosylated hemoglobin; Me, median; Q, quartile; T1DM, type 1 diabetes mellitus; *, statistically significant differences with control group, *p* < 0.05; #, statistically significant differences with Group 1; *p* < 0.05.

**Table 2:** Biochemical blood parameters in men with T1DM (Me, [Q1; Q3]).

Parameters	Group 1	Group 2	Control Group
TC, mmol/L	4.40 [3.80; 4.90]	4.65 [4.10; 5.50] *	4.21 [3.74; 4.58]
TG, mmol/L	1.10 [0.90; 1.90] *	1.20 [0.80; 2.00] *	0.66 [0.47; 0.93]
LDL, mmol/L	2.20 [1.92; 2.70]	2.42 [2.02; 2.83]	2.47 [2.22; 0.99]
HDL, mmol/L	1.30 [1.00; 1.50]	1.25 [1.00; 1.70]	1.23 [1.00; 1.40]
VLDL, mmol/L	0.50 [0.40; 0.86] *	0.54 [0.36; 0.91] *	0.30 [0.21; 0.42]
Creatinine, µmol/L	80.00 [70.00; 90.00] *	80.00 [80.00; 90.00] *	100.90 [87.95; 107.55]
TP, g/L	69.00 [65.00; 74.00] *	76.00 [70.00; 78.00]	76.15 [68.70; 80.45]
Albumin, g/L	43.00 [40.50; 46.00]	44.00 [42.00; 48.00] #	42.84 [39.95; 43.90]
Urine, mmol/L	5.20 [4.10; 5.90]	5.20 [4.30; 6.20]	4.55 [3.85; 5.45]

Abbreviations: TC, total cholesterol; TG, triglycerides; TP, total protein; LDL, low-density lipoproteins; HDL, high-density lipoproteins; VLDL, very-low-density lipoproteins; T1DM, type 1 diabetes mellitus; *n*, number of patients; Me, median; Q, quartile; *, statistically significant differences with the control group, *p* < 0.05; #, statistically significant differences with Group 1, *p* < 0.05.

**Table 3:** Biochemical urine parameters of of men with T1DM (Me, [Q1; Q3]).

Parameters	Group 1	Group 2	Control Group
Creatinine, µmol/L	10.20 [6.36; 17.60]	7.00 [4.61; 14.30] *	14.85 [13.75; 16.30]
Albumin, mg/L	7.10 [3.68; 11.00]	44.30 [24.09; 67.80] *,#	12.70 [7.95; 16.80]
Albumin/creatinine ratio	1.15 [0.30; 1.60]	5.80 [3.80; 8.90] *,#	0.94 [0.55; 1.10]
GFR, mL/min	112.00 [99.00; 120.00] *	101.00 [89.00; 118.00] #	87.00 [78.50; 105.00]

Abbreviations: GFR, glomerular filtration rate; T1DM, type 1 diabetes mellitus; Me, median; Q, quartile; *, statistically significant differences with the control group, *p* < 0.05; #, statistically significant differences with Group 1, *p* < 0.05.

**Table 4:** Oxidative damage product levels in men with T1DM (Me, [Q1; Q3]).

Parameters	Group 1	Group 2	Control Group
CDs, µmol/L	1.68 [1.02; 2.26] *	2.28 [1.86; 3.05] *, #	1.10 [0.87;1.54]
TBARs, µmol/L	0.95 [0.68; 1.51] *	1.21 [0.93; 1.57] *	1.69 [1.28; 2.02]
MGO, mg/L	3.38 [2.92; 3.89] *	3.52 [3.00; 3.96] *	2.14 [1.02; 3.67]
8-OHdG, ng/mL	14.23 [10.97; 16.39]	19.44 [10.32; 25.07] *, #	14.89 [9.24; 18.04]

Abbreviations: CDs, conjugated dienes; TBARs, thiobarbituric acids reactants; T1DM, type 1 diabetes mellitus; MGO, methylglyoxal; Me, median; Q, quartile; 8-OHdG, 8-hydroxy-2-deoxyguanosine; *, statistically significant differences with the control group, *p* < 0.05; #—statistically significant differences with Group 1, *p* < 0.05.

**Table 5:** Total antioxidant status, enzymatic and non-enzymatic antioxidant levels in men with T1DM (Me, [Q1; Q3]).

Parameters	Group 1	Group 2	Control Group
TAS, units	1.14 [1.01; 1.26] *	1.17 [1.03; 1.31]	1.23 [1.15; 1.33]
GPx, units	1099.00 [829.90; 2239.00] *	1029.74 [948.00; 2879.00] *	2224.50 [1944.50; 2492.00]
GR, units	264.10 [167.40; 399.20]	292.75 [225.90; 373.40] *	197.90 [167.75; 268.80]
GSTp, ng/L	3.68 [3.08; 4.52]	4.29 [3.40; 4.90]	3.34 [2.19; 4.28]
SOD-1, units	204.82 [202.97; 205.30]	205.12 [203.59; 205.36]	203.85 [199.56; 210.37]
GSH, mmol/L	2.30 [1.82; 2.68]	2.54 [2.25; 3.37] *, #	2.47 [1.72; 2.66]
GSSG, mmol/L	1.97 [1.54; 2.15]	2.31 [1,94; 2,63] *, #	1.77 [1.47; 1.83]
α-Tocopherol, µmol/L	12.17 [7.81; 14.09] *	12.65 [9.18; 14.75] *	13.84 [11.16; 17.94]
Retinol, µmol/L	1.35 [0.76; 1.92] *	0.81 [0.56; 1.72] *	0.41 [0.36; 0.45]

Abbreviations: GPx, glutathione peroxidase; GR, glutathione reductase; GSTp, glutathione S-transferase; GSH, reduced glutathione; GSSG, oxidative glutathione; SOD-1, superoxide dismutase; TAS, total antioxidant status; T1DM, type 1 diabetes mellitus; Me, median; Q, quartile; *, statistically significant differences with the control group, *p* < 0.05; #, statistically significant differences with Group 1, *p* < 0.05.

**Table 6:** ROC analysis of markers of oxidative damage products and total antioxidant status, enzymatic and non-enzymatic antioxidant levels in men with T1DM Group 1 and Group.

Parameter	AUC		Cut-Off		Sensitivity		Specificity	
	Group 1	Group 2	Group 1	Group 2	Group 1	Group 2	Group 1	Group 2
Oxidative Damage Products								
CDs	**0.755** **(<0.0001)**	**0.714 (0.001)**	>0.91	>1.61	100	85.29	42.86	48.57
TBARs	**0.748 (0.0001)**	**0.708 (0.001)**	<0.98	<0.94	54.30	54.30	96.43	96.40
MGO	**0.720 (0.0033)**	0.554 (0.4375)	>2.84	>3.51	82.86	52.94	71.43	62.86
8-OHdG	0.507 (0.9258)	**0.658 (0.0232)**	>16.86	>16.39	20.00	61.76	64.29	77.14
Enzymatic and Non-Enzymatic Antioxidants								
GPx	**0.741 (0.0004)**	0.580 (0.2500)	<1513.31	>2598.00	65.71	35.29	100.00	85.71
GR	0.620 (0.0894)	0.571 (0.3144)	>320.20	>173.50	40.00	91.18	92.86	31.43
GSTp	0.619 (0.1066)	0.578 (0.2623)	>2.85	>3.92	88.57	58.82	42.86	62.86
SOD-1	0.502 (0.9797)	0.577 (0.2680)	<205.69	>203.04	88.57	88.24	39.29	28.57
GSH	0.510 (0.8911)	**0.667 (0.0108)**	<3.01	>2.11	80.00	94.12	30.00	40.00
GSSG	0.631 (0.0704)	**0.714 (0.0010)**	>1.83	>2.09	62.86	70.59	78.57	71.43
α-Tocopherol	**0.683 (0.0071)**	0.549 (0.4817)	<15.32	>16.49	88.57	17.65	46.43	94.29
Retinol	**0.932** **(< 0.0001)**	0.598 (0.1573)	>0.58	<0.72	82.86	50.00	96.43	80.00
Total Antioxidant Activity								
TAS	**0.686 (0.0052)**	0.574 (0.2851)	<1.09	>1.09	45.71	70.59	89.29	45.71

Abbreviations: AUC, area under curve; CDs, conjugated dienes; TBARs, thiobarbituric acids reactants; T1DM, type 1 diabetes mellitus; MGO, methylglyoxal; 8-OHdG, 8-hydroxy-2-deoxyguanosine; GPx, glutathione peroxidase; GR, glutathione reductase; GSTp, glutathione S-transferase *p*; GSH, reduced glutathione; GSSG, oxidative glutathione; ROC, receiver operating characteristic; SOD-1, superoxide dismutase; TAS, total antioxidant status; Bold indicate a significant parameter.

## Data Availability

The data presented in this study are available in article.
